# Migratory myiasis in a European traveller due to *Hypoderma larvae*

**DOI:** 10.1093/jtm/taac023

**Published:** 2022-02-23

**Authors:** Michelle Verheijden, Luc Laumen, Marlies Mulder, Michel Boshoven, Jeroen Roelfsema, Marjolijn Pronk, Leo G Visser, Marjolijn Wegdam-Blans

**Affiliations:** Department of Family Medicine, University of Maastricht, Maastricht, P.O. Box 616, 6200 MD Maastricht, The Netherlands; Department of Dermatology, Maastricht University Medical Centre+, Maastricht, P.O. Box 616, 6200 MD Maastricht, The Netherlands; Department of Dermatology, Catharina Ziekenhuis, Eindhoven, P.O. Box 1350, 5602 ZA Eindhoven, The Netherlands; Department of Medical Microbiology, Stichting PAMM, Veldhoven, 5504 DL Veldhoven, The Netherlands; Department of Medical Microbiology, Maastricht University Medical Centre+, P.O. Box 5800, 6202 AZ Maastricht, The Netherlands; Department of Medical Microbiology, Stichting PAMM, Veldhoven, 5504 DL Veldhoven, The Netherlands; National Institute for Public Health and the Environment (RIVM), Centre for Infectious Disease Control, P.O. Box 1, 3720 BA Bilthoven, The Netherlands; Department of Internal Medicine, Catharina Ziekenhuis, P.O. Box 1350, 5602 ZA Eindhoven, The Netherlands; Department of Infectious Diseases, Leiden University Medical Center, P.O. Box 9600, 2300 RC Leiden, The Netherlands; Department of Medical Microbiology, Stichting PAMM, Veldhoven, 5504 DL Veldhoven, The Netherlands

A 28-year old otherwise healthy Dutch male presented at the emergency department with fatigue, joint complaints and migratory subcutaneous swellings on back, and legs. Moreover, he reported a parasite coming out of the epidermis of his back. Three months previously, he returned from travelling across South America, South and Southeastern Asia and the Caucasus.

At presentation, routine haematological and biochemical investigations were normal, except for high levels of eosinophils (>10^*^109/l) and an increased erythrocyte sedimentation rate (highest 76 mm/h). Microscopic examination of faces, routine PCRs and serological tests for ascariasis, fascioliasis, filariasis, paragonimiasis, schistosomiasis, strongyloidiasis, toxocariasis and trichinellosis were negative.

**Figure 1:**
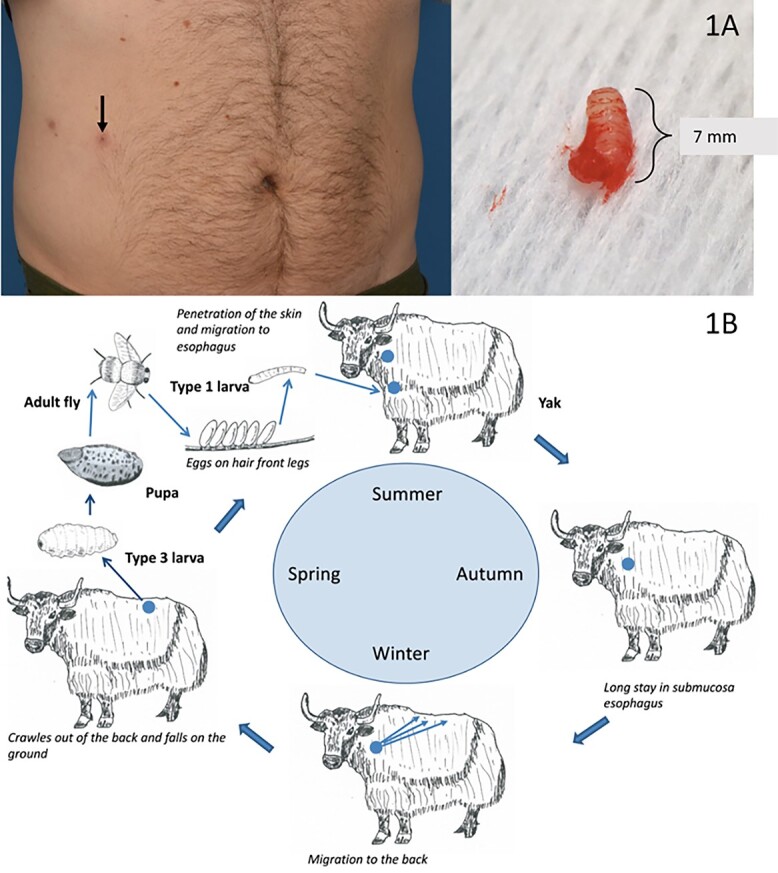
(a) Surgically removed larva removed from a subcutaneous swelling in the patient’s abdomen. (b) Life cycle of the *Hypoderma sinense*/*Hypoderma lineatum* parasite. The adult flies look very similar and their life cycle is identical, but they differ in the host they parasitize: yaks (*H. sinense*) or (domesticated) cattle (*H. lineatum*)

Due to migratory swellings, a *Gnathostoma spinigerum* infestation was suspected and he was treated with albendazole. However, after 2 weeks of treatment, two thick nodules on the patient’s right flank appeared. A white, oval-shaped, 7 mm parasite was surgically removed from one of the nodules (Figure 1A). In addition, serological tests for gnathostomiasis returned negative. Therefore, this diagnosis was rejected and albendazole treatment was ceased.

Finally, molecular examination revealed the diagnosis: *H. sinense* (S1). Further sequencing of mitochondrial targets, however, revealed a large number of mismatches between the specimen and the closest matching *Hypderma sp*, *H. sinense* and *H. lineatum* (S2). Therefore, the specimen could not be assigned to a particular species. Also, the patients’ extensive travel history (across 24 countries) did not help to distinguish between *H. sinense* and *H. lineatum*, since *H. lineatum* is endemic in large parts of Asia and northern Mexico, and *H. sinense* is endemic to Western Himalaya. The patient was treated with ivermectin (12 mg orally once time daily) in three courses of 3 days with several weeks in between. The patients’ condition improved and his blood screen returned to normal.

*Hypoderma sp*. (warble flies), belonging to the Oestridae family, usually cause subcutaneous myiasis in cattle and are generally found in the Northern Hemisphere. *Hypoderma sinense* was first discovered by Pleske in 1926, but soon synonymized with *H. lineatum.* The adult flies look very similar and their life cycle is identical (Figure 1B), although they differ in the host they parasitize.^1^^,^^2^

The diagnostic process was challenging due to the large number of possible parasitic infestations characterized by migratory (sub)cutaneous swelling(s). These infestations include fascioliasis, gnathostomiasis, loaiasis, dirofilariasis, mansolelliasis, paragonimiasis and toxocariasis. However, fascioliasis, paragonimiasis and mansolelliasis are characterized by nodular subcutaneous swelling; loaiasis is only seen at the African continent and *G. spinigerum* larvae cannot mature in humans larger than 3 mm. Sparganosis and dirofilariasis could also be added as possible differential diagnosis typically presenting with subcutaneous migratory swelling(s). However, these infestations were not included in our initial possible differential diagnosis.

Case reports of human infestations with *Hypoderma sp.,* such as *H. lineatum* and *H. sinense* are rare, especially in Europe. In 2014, a case report was published, describing a farmer, who had never travelled outside Italy with an infestation by *H. lineatum*, confirmed by molecular analysis.^3^ Puente et al. (2010) reported on a European with an *H. sinense* infestation, presenting with abdominal pain and, inflammation of the right groin and testicular region after traveling to northern India.^4^ Furthermore, a comment was published (2012) about an *H. sinense* infestation of a German tourist who had developed swellings and pain after traveling to Tibet.^5^

## Supplementary Material

Supplementary_legends_Journal_of_Travel_Medicine_taac023
