# Supplementary material for: Migratory myiasis in a European traveller due to Hypoderma larvae
Source: J Travel Med. 2022 Feb 23;29(5):taac023. doi: 10.1093/jtm/taac023 (PMC9392425; doi:10.1093/jtm/taac023)
Supplement: Supplementary_legends_Journal_of_Travel_Medicine_taac023 [file supplementary_legends_journal_of_travel_medicine_taac023.docx]

**Supplementary legends**

**S1 DNA Sequence Hypoderma Sinense**

Alignment of Cox1 sequence from the larva which was obtained by polymerase chain reaction (PCR) with forward primer cgtaacagctcatgctttt and reverse primer ttgataaagaattggatctcctcctc. GenBank entries EU181169 and EU181167 are from Hypoderma sinense isolate Tianzhu cytochrome c oxidase subunit I (COI) gene and Hypoderma sinense isolate Huangyangzhen COI gene. AF295558 is from Hypoderma lineatum COI gene, a closely related species.

**
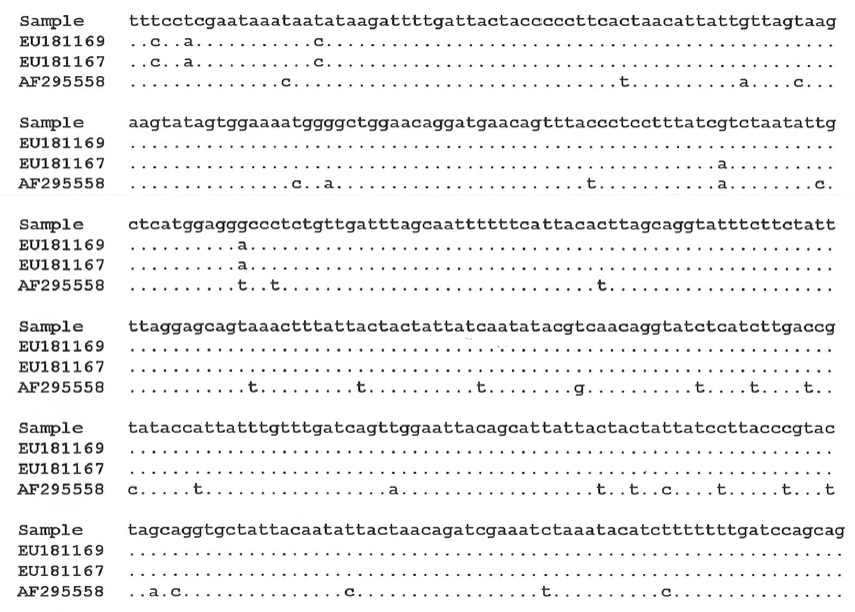
**

**S2 Sequence alignment of larva with Hypoderma species**

PCR was performed with primers based on Hypoderma sinense sequence HM177250 (mt2-f aggtaaagtacctcgtactcaaataaa, mt2-r ttgctggttgatcttcaaattc). Sequences from Hypoderma sp. were retrieved fom GenBank (Accesion numbers HM177245-50), published by Weigl et al. (2010).
